# Clinical and genetic characterization of pediatric patients with progressive familial intrahepatic cholestasis type 3 (PFIC3): identification of 14 novel *ABCB4* variants and review of the literatures

**DOI:** 10.1186/s13023-022-02597-y

**Published:** 2022-12-22

**Authors:** Rong Chen, Feng-Xia Yang, Yan-Fang Tan, Mei Deng, Hua Li, Yi Xu, Wen-Xian Ouyang, Yuan-Zong Song

**Affiliations:** 1 Department of Pediatrics, The First Affiliated Hospital, Jinan University, Guangzhou, 510630 China; 2 Department of Infectious Diseases, Guangzhou Women and Children’s Medical Center, Guangzhou, 510120 China; 3 Department of Hepatopathy, Hunan Children’s Hospital, Changsha, 410007 China

**Keywords:** Progress familial intrahepatic cholestasis type 3 (PFIC3), *ABCB4* gene, Novel variants

## Abstract

**Background:**

Progressive familial intrahepatic cholestasis type 3 (PFIC3) is an autosomal recessive disease caused by pathogenic variants of the gene *ABCB4*. This study aimed to investigate the *ABCB4* genotypic and the clinical phenotypic features of PFIC3 patients.

**Methods:**

The clinical and molecular genetic data of 13 new pediatric patients with PFIC3 as well as 82 reported ones in the PubMed and CNKI databases were collected and analyzed.

**Results:**

The 13 new PFIC3 patients included six females and seven males, and the main presentations were hepatomegaly, splenomegaly, jaundice, and pruritus, as well as increased levels of gamma-glutamyl transpeptidase (GGT). Fourteen new *ABCB4* variants were detected, including eight diagnosed to be likely-pathogenic and six, pathogenic. Among all the 95 PFIC3 cases, hepatomegaly was observed in 85.3% (81/95), pruritus in 67.4% (64/95), splenomegaly in 52.6% (50/95), jaundice in 48.4% (46/95), portal hypertension in 34.7% (33/95) and GGT elevation in 100% (88/88) of the patients. Positive responses at varied degrees to oral ursodeoxycholic acid (UDCA) treatment were observed in 66.1% (39/59) of the patients, among whom 38.5% (15/39) fully recovered in terms of the laboratory changes. Although the condition remained stable in 53 patients (58.9%, 53/90), the clinical outcomes were not promising in the rest 37 cases (41.1%, 37/90), including 7 died, 27 having undergone while another 3 waiting for liver transplantation. A total of 96 *ABCB4* variants were detected in the 95 patients. PFIC3 patients with biallelic null variants exhibited earlier onset ages [10.5 (2, 18) vs. 19 (8, 60) months, p = 0.007], lower UDCA response rate [18.2% (2/11) vs. 77.1% (37/48), p = 0.001], and more unpromising clinical outcomes [80% (12/15) vs. 33.3% (25/75), p = 0.001], compared with those with non-biallelic null variants.

**Conclusions:**

PFIC3 presented with hepatomegaly, pruritus, splenomegaly and jaundice with increased serum GGT level as a biochemistry hallmark. Although varying degrees of improvement in response to UDCA therapy were observed, 41.1% of PFIC3 patients exhibited unfavorable prognosis. *ABCB4* genotypes of biallelic null variants were associated with severer PFIC3 phenotypes. Moreover, the 14 novel variants in this study expanded the *ABCB4* mutation spectrum, and provided novel molecular biomarkers for diagnosis of PFIC3 patients.

**Supplementary Information:**

The online version contains supplementary material available at 10.1186/s13023-022-02597-y.

## Introduction

Progressive familial intrahepatic cholestasis (PFIC) included a group of rare autosomal recessive diseases caused by pathogenic variants of the genes encoding proteins related to the formation and transfer of bile acids in the liver [1]. The PFIC patient's onset varied from the neonatal period to early adulthood, which usually developed fibrosis and end-stage liver disease before adulthood [2]. Based on different causative genes, PFIC could be divided into types 1–6, with *ATP8B1*, *ABCB11*, *ABCB4*, *TJP2*, *NR1H4*, and *MYO5B* being the causative gene, respectively [3].

The gene *ABCB4* causing PFIC3 (OMIM # 602347) was located on chromosome 7q21, which encoded a liver-specific canalicular transporter, the Multi-Drug Resistant 3 (MDR3) protein [4]. MDR3 translocated phosphatidylcholine from the inner to the outer leaflet of the canalicular membrane, resulting phosphatidylcholine efflux into the bile [5, 6]. In the aqueous environment of bile, phospholipids form mixed micelles with cholesterol and bile acids, thereby preventing the formation of cholesterol gallstones and the detergent action of free bile acids which was injurious to cholangiocyte membrane [7, 8]. Typical clinical features of PFIC3 included jaundice, pruritus, hepatomegaly, and splenomegaly, which could progress to cirrhosis and liver failure before adulthood. Thus far, due to the lack of pathognomonic clinical symptoms or signs, the definitive diagnosis of PFIC3 relied on the *ABCB4* genetic analysis [9].

In the recent years, the clinical application of molecular genetic techniques facilitated the timely diagnosis of PFIC patients, and an increasing number of PFIC3 patients were reported around the world [10–15]. However, the molecular and clinical characteristics of this condition, generally as a rare liver disease, remained yet far from being completely understood. This study analyzed the phenotypic and genotypic features of PFIC3 patients by reporting 13 new pediatric patients and reviewing the relevant literatures.

## Methods

### Subjects and ethical approval

The research subjects in this study included 13 pediatric patients including seven males and six females from 12 unrelated families. The clinical data of the 13 patients were collected for analysis, including their ages, genders, history, clinical presentations, laboratory changes, treatment and outcomes. Most the data were collected from the medical record databases in the participating hospitals, with partial data from other hospitals being provided by patients' parents at their referrals to our clinics.

This study was approved by the Medical Ethics Committee of the First Affiliated Hospital, Jinan University, and written informed consents were signed by the parents of all patients before this study.

### Genetic analysis

Genomic DNA was obtained from peripheral blood according to standard procedures, and all patients underwent next-generation sequencing (NGS) of the targeted or whole exomes, to explore the underling genetic causes. *ABCB4* variants detected were then verified by Sanger sequencing. The sequencing results were aligned with the *ABCB4* gene sequence (ENST00000649586.2), which was available at Ensembl Genome Browser (www.ensembl.org). The variant nomenclature was in agreement with current guidelines of the Human Genome Variation Society (http://www.hgvs.org/rec.html).

### Pathogenicity evaluation

All the variants were classified according to the American College of Medical Genetics and Genomics (ACMG) standards and guidelines.

The allele frequencies of the identified variants were collected from the 1000 Genomes Project (https://www.internationalgenome.org), the Genome Aggregation Database (gnomeAD, http://gnomad-sg.org), the Human Gene Mutation database (http://www.hgmd.cf.ac.uk/ac/index.php), and all relevant literatures in database of PubMed (https://pubmed.ncbi.nlm.nih.gov).

Conservation of mutated amino acids was analyzed by comparatively aligning the amino acid sequences of *ABCB4* orthologs collected from the Ensembl Genome Browse. The 20 primate homologous proteins include human, angola colobus, tarsier, chimpanzee, bonobo, bushbaby, black snub-nosed monkey, gorilla, drill, capuchin, gelada, gibbon, macaque, olive baboon, mouse lemur, golden snub-nosed monkey, sooty mangabey, coquerel’s sifaka, pig-tailed macaque, and bolivian squirrel monkey.

The pathogenicity of the novel missense variants was predicted by using the three online programs PolyPhen-2 (http://genetics.bwh.harvard.edu/pph2), Mutation Taster (http://www.mutationtaster.org) and PROVEAN (http://provean.jcvi.org/seq_submit.php). PolyPhen-2 analysis identified variant as probably damaging if the probability was > 0.85, and possibly damaging if the probability was > 0.15. Mutation Taster value close to 1 indicated a high security of the prediction. PROVEAN predicted a variant as “deleterious” if the prediction score was < − 2.5. Moreover, the online bioinformatics tools NNspl (http://www.fruitfly.org/seq_tools/splice.html) and Human Splicing Finder (http://www.umd.be/HSF3/HSF.shtml) were used to assess the potential of splicing-site variants to disrupt normal splicing.

### Review of the literatures

Electronic databases including PubMed and CNKI (https://www.cnki.net) were retrieved by using the keywords “*ABCB4*” and “PFIC3”. The genotypic and phenotypic data of the pediatric patients with clear molecular genetic diagnosis and detailed clinical information were collected and analyzed.

### Statistical analysis

Data were analyzed with the use of IBM SPSS Statistics 26 software (IBM, Armonk, NY, USA). Normally distributed data were expressed as mean ± SD and then compared using Student’s t-test. Data of skewed distribution were presented as the median values (P25, P75), and comparisons were conducted by means of Mann–Whitney U-test. Categorical variables were expressed as percentages, and statistical differences were compared by Chi-Square or Fisher’s exact test. Statistical significance was set at p < 0.05.

## Results

### Clinical and genetic characteristics of the 13 new PFIC3 patients

Table 1 summarized the clinical information of the 13 new PFIC3 patients from 12 unrelated families. The ages of symptom onset were 36 (8, 67) months. As the commonest clinical presentation in this cohort, hepatomegaly was observed in 13 patients, followed by splenomegaly in 11, jaundice in seven, pruritus in four cases. The biochemistry hallmark was markedly increased levels of gamma-glutamyl transpeptidase (GGT) in all patients. On the last follow up, two patients demonstrated unfavorable outcomes: one was waiting for liver transplantation due to hepatic decompensation and one had died of liver failure. Nine patients were alive with stable condition, and the rest two lost contact.

**Table 1:** Clinical information of the 13 new PFIC3 patients

Patient No	Sex	Family history	Age at onset	Clinical presentations	Age	Anthropometry and laboratory changes at first referral									Pathologic Features	Outcomes at the last follow up
						Wt (kg)	Ht (cm)	ALT	AST	GGT	TBIL	DBIL	TBA	25(OH)D		
1	Female	No	4.2Y	Pruritus, splenomegaly, hepatomegaly, jaundice	5Y	17 (− 0.6SD)	105 (− 1.2SD)	277	232	589	48.8	32.9	88.3	4.87	NA	Aged 8Y, awaiting LT
2	Female	Elder sister of patient 3	10.8Y	Jaundice, hepatomegaly, splenomegaly, gastrointestinal bleeding, portal hypertension	11.3Y	NA	NA	63	149	302	115.4	109.4	125.3	NA	NA	Died of liver failure at the age 12Y
3	Male	Younger brother of patient 2	7.2Y	Hepatomegaly, splenomegaly	8.5Y	27.5 (− 0.3SD)	131 (− 0.3SD)	109	88	95	3.7	2	7.5	NA	NA	Loss of follow-up
4	Female	No	3Y	Hepatomegaly, splenomegaly, failure to thrive	6.7Y	17 (− 2.8SD)	109 (− 2.9SD)	83	56	72	15.5	6.6	93	28	Nodular cirrhosis	Aged 9Y, alive
5	Female	Elder sister and brother died of liver failure	1 M	Jaundice, hepatomegaly	2 M	5.7 (+ 0.8SD)	58.8 (+ 0.6SD)	43	35	637	67.2	20	154.4	20.7	NA	Aged 4 M, alive
6	Male	Elder brother with PFIC3, LT at 9y because of liver failure	3D	Jaundice, hepatomegaly, splenomegaly, pruritus	6Y	23.5 (+ 0.8SD)	116 (− 0.4SD)	381	173	307	222	100.8	63.3	5.36	NA	Aged 7Y, alive
7	Female	Elder sister died of intracranial hemorrhage	6 M	Hepatomegaly, splenomegaly, jaundice	8 M	7.5 (-1.3SD)	68 (-1.5SD)	120	175	108	67.3	56.4	280.2	NA	NA	Aged 1.3Y, alive
8	Male	No	1Y	Hepatomegaly	1.6Y	12.5 (+ 0.9SD)	80.4 (-0.7SD)	184	130	102	6.1	3.8	98.6	27.6	NA	Aged 2Y, alive
9	Female	No	3Y	Hepatomegaly, splenomegaly, jaundice, discolored stools	3Y	12.5 (− 1.3SD)	94 (− 0.4SD)	186	207	202	17.1	3.6	148.2	NA	NA	Aged 5.1Y, alive
10	Male	Elder brother died of liver failure	6.2Y	Hepatomegaly, splenomegaly	6.2Y	21.8 (− 0.5SD)	115.6 (+ 0.2SD)	127	125	354	11.7	6.5	22.2	NA	NA	Aged 8.3Y, alive
11	Male	Mother with intrahepatic biliary stones. Elder brother and sister died of liver failure	5Y	Pruritus, hepatomegaly, splenomegaly, failure to thrive	6Y	16.1 (− 2.8SD)	106.5 (− 2.5SD)	228	99	136	21.6	7.4	276	NA	Liver fibrosis	Aged 9.4Y, alive
12	Male	No	10 M	Jaundice, hepatomegaly, splenomegaly	2.5Y	14 (− 0.4SD)	95 (− 1SD)	30	85	206	50.1	17.1	114.5	NA	NA	Loss of follow-up
13	Male	Mother with ICP	4.3Y	Hepatomegaly, splenomegaly, pruritus	5Y	18 (− 0.5SD)	107 (− 1SD)	156	145	197	12.9	8.3	108.6	NA	Ductal proliferation and inflammatory infiltration	Aged 5.4Y, alive

Reference ranges: ALT: (5–40U/L); AST: (5–40U/L); GGT: (8–50U/L); Tbil: (5.1–23 μmol/L); Dbil: (0.6–6.8 μmol/L); TBA: (0–10 μmol /L); 25(OH)D: (≥ 20 ng/ml). Among the five patients with 25(OH)D analyzed, two exhibited vitamin D deficiency(< 20 ng/ml)

*Y* year; *M* month; *D* day; *ICP* intrahepatic cholestasis of pregnancy; *Wt* weight; *Ht* height; *ALT* alanine aminotransferase; *AST* aspartate aminotransferase; *GGT* γ-glutamyl transpeptidase; *Tbil* total bilirubin; *Dbil* direct bilirubin; *TBA* total bile acids; *25(OH)D* 25-hydroxyvitamin D; *NA* not available; *LT* liver transplantation.

**Fig. 1:**
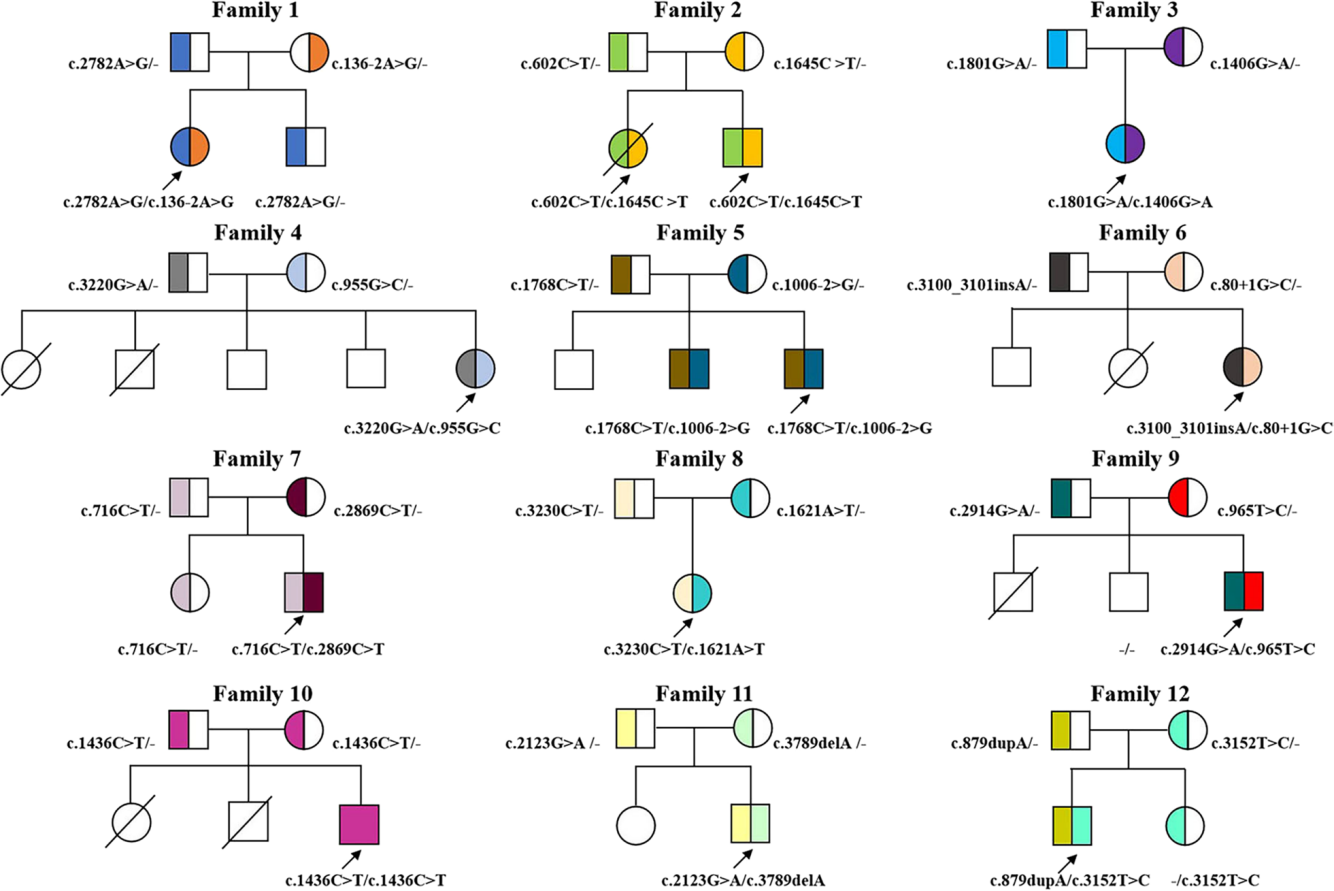
*ABCB4* genotypes of the 13 new PFIC3 patients from 12 unrelated families. The arrows indicated the probands in each family, and the deceased individuals were shown with a slash. Circles and squares represented females and males, respectively. The different *ABCB4* variants were illustrated in different colors in this figure

All the 13 patients were either homozygous or compound heterozygous for *ABCB4* variants (Additional file 1: Fig. S1). As listed in Fig. 1, a total of 23 *ABCB4* variants were detected in the 13 new PFIC3 patients, and 14 variants were not detected in any PFIC3 patients previously, including eight missense c.2782A > G(p.Arg928Gly), c.1645C > T(p.Arg549Cys), c.1801G > A (p.Ala601Thr), c.1406G > A(p.Arg469Lys), c.716C > T(p.Ser239Leu), c.3230C > T(p.Thr1077Met), c.2914G > A(p.Asp972Asn), and c.965 T > C(p.Leu322Pro), three frameshift c.3100_3101insA(p.Ile1034Asnfs*4), c.3789delA(p.Gly1264Alafs*38) and c.879dupA(p.Ala294Serfs*62), two splicing-site c.136-2A > G and c.80 + 1G > C, as well as one nonsense variant(s) c.2123G > A(p.Trp708*).

**Fig. 2:**
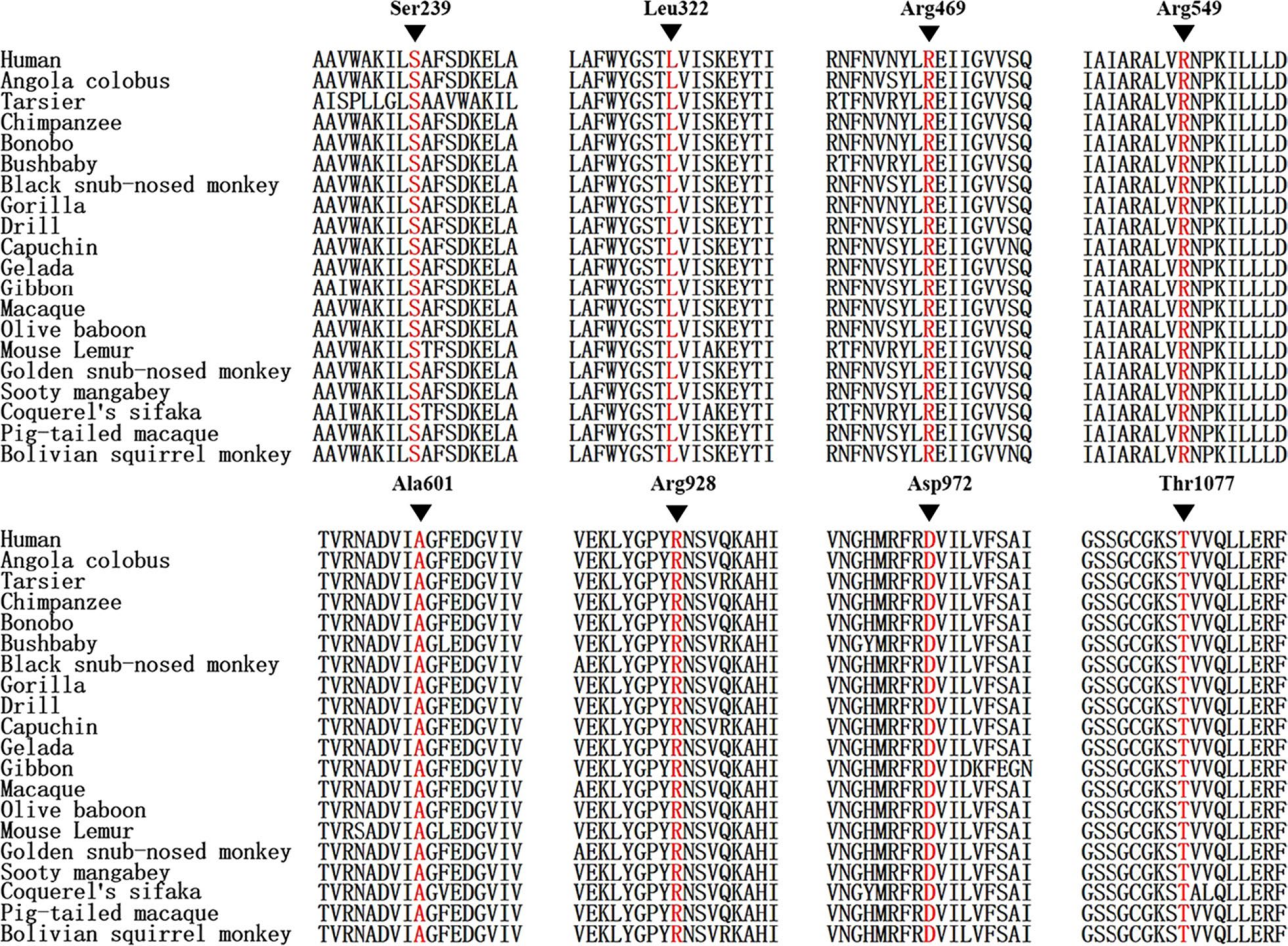
Comparative alignment of the homologous peptides affected by the eight novel missense variants in 20 primate species. The eight novel missense variants all affected a highly conserved amino acid residue of MDR3 protein

The variants c.1801G > A(p.Ala601Thr) and c.3230C > T(p.Thr1077Met) were included in the database gnomeAD with the allele frequencies of 0.007962‰ and 0.1991‰, respectively, while the rest 12 novel variants have neither been reported in any official literatures nor included in any variant databases, to the best of our knowledge. The amino acid sequences of the homologous peptides in a total of 20 primates were aligned comparatively, and the results showed the eight novel missense variants were localized in highly conserved regions of all the 20 primate homologous proteins (Fig. 2).

In the study, six out of the 14 novel variants were classified to be pathogenic, and remaining eight as likely pathogenic, according to the ACMG standards. The relevant evidences were listed in detail in Table 2.

**Table 2:** Novel *ABCB4* variants and the pathogenicity classification

Patient No	Nucleotide changes	Amino acid changes	Variant types	ACMG evidences	ACMG classification	Allele Frequency		In silico verdict				
						1000 Genomes	gnomAD	Mutation Taster	PolyPhen-2	PROVEAN	HSF	NNsplice
1	c.2782A > G	p.Arg928Gly	Missense	PM2 + PM3 + PP1-4	Likely pathogenic	NI	NI	D	ProbD	D	–	–
1	c.136-2A > G	–	Splicing	PVS1 + PM2 + PP1 + PP3 + PP4	Pathogenic	NI	NI	–	–	–	S	S
2/3	c.1645C > T	p.Arg549Cys	Missense	PM2 + PM3 + PP1-4	Likely pathogenic	NI	NI	D	ProbD	D	–	–
4	c.1801G > A	p.Ala601Thr	Missense	PM2 + PP1-4	Likely pathogenic	NI	0.007962‰	D	PossD	D	–	–
4	c.1406G > A	p.Arg469Lys	Missense	PM2 + PP1-4	Likely pathogenic	NI	NI	D	ProbD	D	–	–
7	c.3100_3101insA	p.Ile1034Asnfs*4	Frameshift	PVS1 + PM2 + PP1 + PP4	Pathogenic	NI	NI	–	–	–	–	
7	c.80 + 1G > C	–	Splicing	PVS1 + PM2 + PP1 + PP3 + PP4	Pathogenic	NI	NI	–	–	–	S	S
8	c.716C > T	p.Ser239Leu	Missense	PM2 + PM3 + PP1-4	Likely pathogenic	NI	NI	D	ProbD	D	–	–
9	c.3230C > T	p.Thr1077Met	Missense	PM2 + PM3 + PP1-4	Likely pathogenic	NI	0.1991‰	D	ProbD	D	–	–
10	c.2914G > A	p.Asp972Asn	Missense	PM2 + PP1-4	Likely pathogenic	NI	NI	D	B	N	–	–
10	c.965 T > C	p.Leu322Pro	Missense	PM2 + PP1-4	Likely pathogenic	NI	NI	D	ProbD	D	–	–
12	c.2123G > A	p.Trp708*	Nonsense	PVS1 + PM2 + PP1 + PP4	Pathogenic	NI	NI	–	–	–	–	–
12	c.3789delA	p.Gly1264Alafs*38	Frameshift	PVS1 + PM2 + PP1 + PP4	Pathogenic	NI	NI	D	–	–	–	–
13	c.879dupA	p.Ala294Serfs*62	Frameshift	PVS1 + PM2 + PP1 + PP4	Pathogenic	NI	NI	–	–	–	–	–

According to the ACMG criteria [35]: PVS1, null variant (nonsense, frameshift, canonical ± 1 or 2 ss, etc.) in a gene where loss of function is a known mechanism of disease; PM2, absent from controls (or at extremely low frequency if recessive) absent from controls in 1000 Genomes Project, or gnomAD; PM3, for recessive disorders, detected in trans with a pathogenic variant; PP1, co-segregation with disease in multiple affected family members in a gene definitively known to cause the disease; PP2, missense variant in a gene that has a low rate of benign missense variation and where missense variants are a common mechanism of disease; PP3, multiple lines of computational evidence support a deleterious effect on the gene or gene product; PP4, patient’s phenotype or family history is highly specific for a disease with a single genetic etiology; -, not applicable; *NI* not included; *D* disease causing/deleterious; *PossD* possibly damaging; *ProbD* probably damaging; *B* benign; *N* neutral/not affecting; *S* splicing potential alteration of splicing, *HSF* human splicing finder

### Findings of literature review

The clinical and molecular genetic data of 82 pediatric PFIC3 patients, who were definitely diagnosed with biallelic *ABCB4* variants and had relatively complete clinical information in 20 official literatures [11–13, 15–31], were summarized in Additional file 2: Table S1. Together with the 13 new PFIC3 patients in this study, a total of 95 patients (38 females, 39 males and 18 gender undescribed) were in-depth analyzed in terms of the phenotypic and genotypic features.

The ages of symptom onset were 18 (7, 50) months. Hepatomegaly was observed in 85.3% (81/95), pruritus in 67.4% (64/95), splenomegaly in 52.6% (50/95), jaundice in 48.4% (46/95), portal hypertension in 34.7% (33/95) and failure to thrive (14.7%, 14/95) of all the cases. In addition, gastrointestinal bleeding, discolored stools, gallstone, abdominal distension, ascites, cholecystitis, and reduced bone density were also observed some patients. Regarding serum biochemistry, 88 patients (100%, 88/88) exhibited increased levels of GGT. Besides, the elevation of aspartate transaminase (ALT), alanine transaminase (AST), total bilirubin (TBil), direct bilirubin (DBil), total bile acids (TBA) was observed in 91.9% (68/74), 98.4% (61/62), 45.5% (30/66), 45.3% (24/53) and 93.8% (45/48) of the patients, respectively.

Positive responses at varied degrees to oral ursodeoxycholic acid (UDCA) treatment were observed in 66.1% (39/59) of the patients, of whom 38.5% (15/39) fully recovered in terms of the laboratory changes. In clinical outcome analysis on the last follow-up, five out of all 95 patients were excluded due to loss of contact; the condition remained stable in 53 patients (58.9%, 53/90), while the clinical outcomes were not promising in the rest 37 cases (41.1%, 37/90), including 7 died, 27 having undergone while another 3 waiting for liver transplantation.

**Fig. 3:**
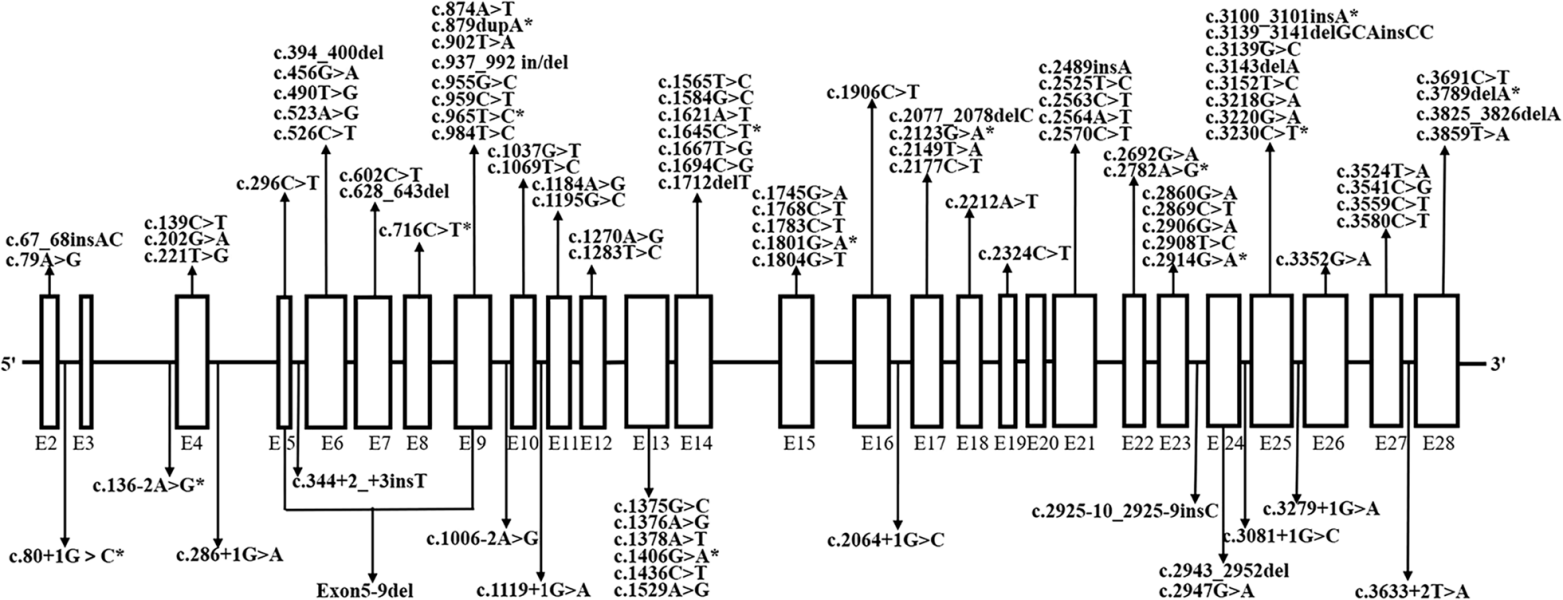
The distribution of the 96 *ABCB4* variants detected in the 95 PFIC3 patients in this study. The novel *ABCB4* variants identified in this study were marked with asterisks. E2-E28 represented the 27 encoding *ABCB4* exons

Among the 95 PFIC3 patients, a total of 96 different *ABCB4* variants were detected, and missense variants 60.4% (58/96) was on top of the list, followed by frameshift (14.6%, 14/96), nonsense (11.5%, 11/96), splicing-site (12.5%, 12/96), exons deletion (1%, 1/96), as summarized in Fig. 3.

### Genotype–phenotype correlation

In this study, the frameshift, nonsense, canonical ± 1 or 2 splicing-site variants and exons deletion were defined as null variants, while missense and non-canonical splicing-site, as non-null variants, according to the ACMG standards. The *ABCB4* phenotypes of the 95 PFIC3 patients were categorized into two groups: biallelic null variants (n = 18) and non-biallelic null variants (n = 77). It was found that PFIC3 patients with biallelic null variants exhibited earlier onset ages [10.5 (2, 18) vs. 19 (8, 60) months, p = 0.007], lower UDCA response rate [18.2% (2/11) vs. 77.1% (37/48), p = 0.001)], and more unpromising clinical outcomes [80% (12/15) vs. 33.3% (25/75), p = 0.001], compared with those with non-biallelic null variants genotypes.

## Discussion

This study described 13 new PFIC3 patients from 12 unrelated families, and among the 23 *ABCB4* variants detected, 14 were not reported previously in any official literatures, including eight missense, three frameshift, two splicing-site, and one nonsense variant(s). The two frameshift variants c.3100_3101insA(p.Ile1034Asnfs*4) and c.879dupA(p.Ala294Serfs*62) as well as the nonsense variant c.2123G > A(p.Trp708*) introduced premature stop codons in the MDR3 residues 1037, 355 and 708, respectively. Another frameshift variant c.3789delA(p.Gly1264Alafs*38) led to prolongation of the MDR3 molecule though the loss of the original stop codon. The canonical splicing-site variants c.136-2A > G and c.80 + 1G > C disrupted the normal acceptor or donor splicing site in the *ABCB4* introns 2 and 3, respectively. According to the ACMG standards and guidelines, the six novel variants above were all null *ABCB4* variants, and was diagnosed to be pathogenic, with the relevant evidences listed in Table 2.

The remaining eight novel missense variants were all absent or included rather rarely included in public databases (PM2), and among them, c.2782A > G(p.Arg928GlyA), c.1645C > T(p.Arg549Cys), c.716C > T(p.Ser239Leu), and c.3230C > T(p.Thr1077Met) proved to be in trans with a previously-reported pathogenic variant by testing parents (PM3) [11, 21]. All the novel missense variants cosegregated with the PFIC3 phenotype in this study (PP1). It was well-known that *ABCB4* missense variants were a common mechanism for PFIC3 development (PP2) [21, 24, 27, 32–34]. In silico prediction suggested them to be disease-causing/ deleterious/possibly damaging/probably damaging, and with the involved amino acid residues all being highly conserved among 20 primates (PP3). Moreover, the biochemical and clinical presentations of the ten patients were quite specific for PFIC3 (PP4). Although in vitro or in vivo functional analysis was not performed due to technical limitation, the evidences above rendered the eight novel missense variants all “likely pathogenic” and supported the diagnosis of PFIC3 in the patients, since according to the ACMG standards, a variant classified as likely pathogenic typically has sufficient evidence that a health-care provider can use the molecular testing information in clinical decision making when combined with other evidence of the disease in question [35].

The MDR3 protein was primarily expressed in the liver, functioning as a floppase that translocated specifically phosphatidylcholine from the inner to the outer leaflet of the hepatocytes canalicular membranes [36]. Phosphatidylcholine was solubilized by canalicular bile salts to form mixed micelles, therefore protecting the biliary tree from exposure to toxic and detergent effects of bile salts [37]. The *ABCB4* variants in this study impaired the floppase function of the MDR3 protein, and thus the depletion of phosphatidylcholine and elevation of hydrophobic bile acids in the biliary tubules damaged the integrity of the cholangiocyte membrane, leading to the development of intrahepatic cholestasis and presenting as hepatomegaly, pruritus, splenomegaly, jaundice and portal hypertension. Besides, low phosphatidylcholine levels would be expected to destabilize micelles and promote lithogenicity of bile with crystallization of cholesterol, which could facilitate liver damage by obstructing small bile ducts [38]. This could explain the occurrence of gallstones in a small number of children with PFIC3 as shown in Additional file 2: Table S1.

In this study, all PFIC3 patients exhibited increased serum GGT levels, constituting a biochemistry hallmark of this condition. Serum GGT was deemed to be mainly of hepatobiliary origin and has been used as a “liver test” for decades [39]. The reasons for elevated GGT values in those with hepatobiliary disease included de novo synthesis, release of membrane-bound GGT (by detergent effects of bile acids), regurgitation of bile into the blood stream, and change in permeability or destruction of biliary epithelial cells [40]. Due to the impaired MDR3 function, PFIC3 patients lacked phosphatidylcholine in the bile to form micelles, and thus the very detergent bile liberated GGT from the canalicular membrane, giving rise to cholangitis with high serum GGT activity [41, 42].

At present, medical treatment was the first line of therapy offered to PFIC3 patient [9, 43], and the major goal of medical treatment was to relieve symptoms, improve the nutritional status, and to treat or prevent complications due to cirrhosis and portal hypertension [38]. UDCA was the most common medicine in patients with PFIC3 [44], and the PFIC3 patients with residual phosphatidylcholine secretion and MDR3 expression, especially those with missense variants, responded to UDCA in 70% of cases [45], and even in those with cirrhosis, UDCA could delay PFIC3 progression [12]. In this study, 66.1% (39/59) of the patients had positive responses to oral UDCA, of whom 38.5% (15/39) fully recovered in terms of the laboratory changes. This was not surprising since UDCA had multiple mechanisms of action in cholestatic disorder including protection of cholangiocytes against cytotoxicity of hydrophobic bile acids, stimulation of hepatobiliary secretion of hydrophobic bile acids, inhibition of liver cell apoptosis, as well as anti-inflammation and immunomodulation [46, 47].

Although most PFIC3 patients showed varying degrees of improvement in response to UDCA therapy, unfavorable prognosis was observed in some cases. Actually, this condition was progressive in the majority of affected patients, and carried a high risk of developing cirrhosis and liver failure during the first 2 decades of life [48]. In this study, 41.1% (37/90) of PFIC3 patients had a poor prognosis, including 7 died, 27 having undergone while another 3 waiting for liver transplantation. On the last follow up, the condition remained stable in 53 patients (58.9%, 53/90), but their long-term prognoses were still uncertain, which needed to be followed up. So far, like other end-stage liver disease, liver transplantation remained the last resort in patients unresponsive to medical treatment [49]. Nevertheless, the lack of donor liver organ and lifelong burden of immunosuppressive therapy restricted the treatment option for this devastating condition [50].

*ABCB4* variants exhibited remarkable heterogeneity, and the extent to which they impaired MDR3 floppase activity determined the course and outcome of the PFIC3 patients [21]. Depending on whether they affected the traffic, activity, or stability of the protein, *ABCB4* variants could be classified as follows: (I) defective synthesis, mainly nonsense and frameshift variants, (II) affect protein maturation, (III) with little or no effect on protein maturation but defective proteins, (IV) affect the stability and (V) variants without detectable effects, providing the strong basis for the development of genotype-based therapies for PFIC3 [24]. This study found that PFIC3 patients with biallelic null variants exhibited earlier onset ages, lower UDCA response rate and more unpromising clinical outcomes, which clearly indicated that null *ABCB4* variants were associated with severer PFIC3 phenotypes. Understanding the genotype–phenotype correlation contributed to the prediction of prognosis and provided additional guidance to physicians and patients about the likely disease course [15].

## Conclusions

PFIC3 presented with hepatomegaly, pruritus, splenomegaly and jaundice with increased serum GGT level as a biochemistry hallmark. Although varying degrees of improvement in response to UDCA therapy were observed, 41.1% of PFIC3 patients exhibited unfavorable prognosis. *ABCB4* genotypes of biallelic null variants were associated with severer PFIC3 phenotypes. Moreover, the 14 novel variants in this study expanded the *ABCB4* variant mutation spectrum, and provided novel molecular biomarkers for the definite diagnosis of PFIC3 patients.

## Supplementary Information

**Additional file 1**.** Figure S1**.* ABCB4* genotypes of the 12 unrelated families on Sangersequencing or next generation sequencing. Arrows indicated the mutations. Since Sangervalidation through forward sequencing or reverse sequencing, the base of the peak map maybe the reverse complemen tation sequence of the base detected.

**Additional file 2**.** Table S1**. Clinical and molecular genetic data of previously reported 82 PFIC3 patients.

## Data Availability

The datasets generated and analyzed for this study were available from the corresponding author on reasonable request.

## Abbreviations

PFIC3: Progressive familial intrahepatic cholestasis type 3
GGT: Gamma-glutamyl transpeptidase
UDCA: Ursodeoxycholic acid
MDR3: Multi-drug resistant 3
NGS: Next-generation sequencing
ACMG: American College of Medical Genetics and Genomics
ALT: Aspartate transaminase
AST: Alanine transaminase
TBil: Total bilirubin
DBil: Direct bilirubin
TBA: Total bile acids
