# Supplementary figures and images for: Clinical and genetic characterization of pediatric patients with progressive familial intrahepatic cholestasis type 3 (PFIC3): identification of 14 novel ABCB4 variants and review of the literatures
Source: Orphanet J Rare Dis. 2022 Dec 22;17:445. doi: 10.1186/s13023-022-02597-y (PMC9773540; doi:10.1186/s13023-022-02597-y)

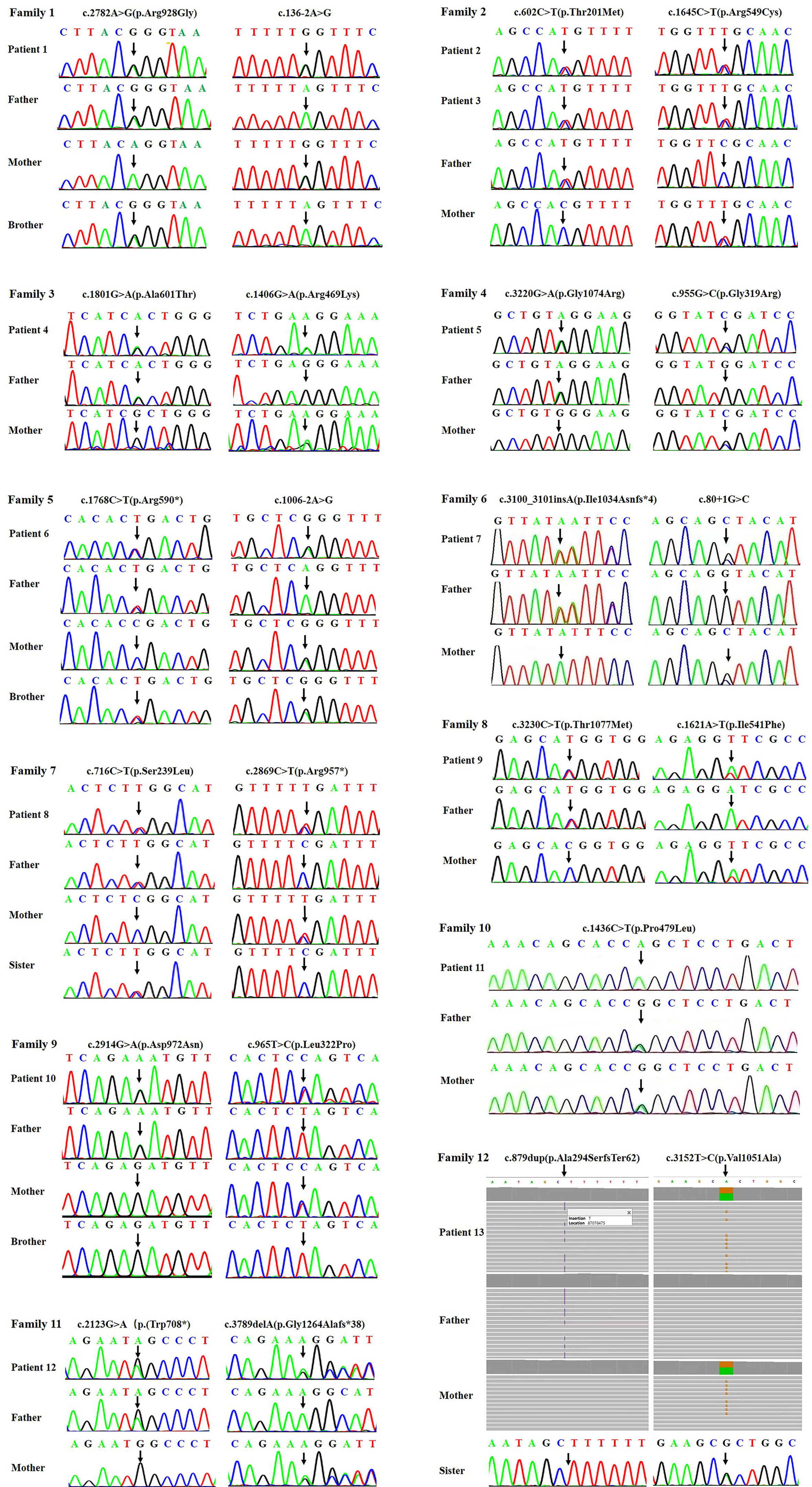

Supplement: Supplementary file 1 — Additional file 1. Figure S1. ABCB4 genotypes of the 12 unrelated families on Sangersequencing or next generation sequencing. Arrows indicated the mutations. Since Sangervalidation through forward sequencing or reverse sequencing, the base of the peak map maybe the reverse complemen tation sequence of the base detected. [file 13023_2022_2597_MOESM1_ESM.jpg]
