# Supplementary material for: Clinical and genetic characterization of pediatric patients with progressive familial intrahepatic cholestasis type 3 (PFIC3): identification of 14 novel ABCB4 variants and review of the literatures
Source: Orphanet J Rare Dis. 2022 Dec 22;17:445. doi: 10.1186/s13023-022-02597-y (PMC9773540; doi:10.1186/s13023-022-02597-y)
Supplement: Supplementary file 2 — Additional file 2. Table S1. Clinical and molecular genetic data of previously reported 82 PFIC3 patients. [file 13023_2022_2597_MOESM2_ESM.docx]

**Supplementary Table 1. Clinical and molecular genetic data of previously reported 82 PFIC3 patients.**

| **Patient No.** | **Sex** | **Age of onset** | **Clinical presentations** | **ABCB4 genotypes** | **ALT** | **AST** | **GGT** | **TBIL** | **DBIL** | **TBA** | **UDCA response** | **Outcome** | **References** |
| --- | --- | --- | --- | --- | --- | --- | --- | --- | --- | --- | --- | --- | --- |
| 1 | Female | 1Y | Pruritus, jaundice, hepatomegaly, splenomegaly, portal hypertension | exons 5-9 del/ exons 5-9 del | Elevated | Elevated | Elevated | Elevated | Elevated | Elevated | NA | Died(8Y) | Chen HL et al. 2001 |
| 2 | NA | 2.5Y | Jaundice, hepatomegaly, portal hypertension | c.79A>G(p.Ser27Gly)/ c.79A>G(p.Ser27Gly) | NA | NA | Elevated | NA | NA | NA | Negative | LT(5Y) | Jacquemin et al. 2001 |
| 3 | NA | 14M | Jaundice, hepatomegaly, pruritus, portal hypertension | c.79A>G(p.Ser27Gly)/ c.79A>G(p.Ser27Gly) | NA | NA | Elevated | NA | NA | NA | NA | LT(5Y) |  |
| 4 | NA | 3M | Jaundice, hepatomegaly, portal hypertension | c.394_400del(p.Tyr132Lysfs*29)/ c.394_400del(p.Tyr132Lysfs*29) | NA | NA | Elevated | NA | NA | NA | Negative | LT(3.5Y) |  |
| 5 | NA | 1Y | Pruritus, discolored stools, jaundice, hepatomegaly, portal hypertension | c.1712delT(p.Val571Aspfs*15)/ c.1712delT(p.Val571Aspfs*15) | NA | NA | Elevated | NA | NA | NA | Negative | LT(6Y) |  |
| 6 | NA | 8M | Hepatomegaly, splenomegaly, pruritus, portal hypertension | c.2943_2952del(p.Phe982Trpfs*3)/ c.2947G>A(p.Gly983Ser) | NA | NA | Elevated | NA | NA | NA | Intermediate | Alive(18Y) |  |
| 7 | NA | 2M | Jaundice, discolored stools, hepatomegaly, portal hypertension | c.1906C>T(p.Gln636*)/ c.1906C>T(p.Gln636*) | NA | NA | Elevated | NA | NA | NA | NA | LT(2.5Y) |  |
| 8 | NA | 1M | Jaundice, hepatomegaly, discolored stools, portal hypertension, gallstone | c.1906C>T(p.Gln636*)/ c.1906C>T(p.Gln636*) | NA | NA | Elevated | NA | NA | NA | Negative | LT(4Y) |  |
| 9 | NA | 8M | Pruritus, jaundice, hepatomegaly, portal hypertension | c.2869C>T(p.Arg957*)/ c.2869C>T(p.Arg957*) | NA | NA | Elevated | NA | NA | NA | Negative | LT(9Y) |  |
| 10 | NA | 3M | Jaundice, pruritus, hepatomegaly, portal hypertension | c.1037G>T(p.Ser346Ile)/ c.1037G>T(p.Ser346Ile) | NA | NA | Elevated | NA | NA | NA | Negative | LT(10Y) |  |
| 11 | NA | 5Y | Hepatomegaly, splenomegaly, jaundice, portal hypertension, gallstone | c.1184A>G(p.Glu395Gly)/ c.1184A>G(p.Glu395Gly) | NA | NA | Elevated | NA | NA | NA | NA | LT(12.5Y) |  |
| 12 | NA | 1Y | Pruritus, hepatomegaly, portal hypertension | c.1621A>T(p.Ile541Phe)/ c.1621A>T(p.Ile541Phe) | NA | NA | Elevated | NA | NA | NA | Negative | LT(5Y) |  |
| 13 | NA | 5M | Hepatomegaly, discolored stools, pruritus, jaundice, portal hypertension | c.1667T>G(p.Leu556Arg)/ c.1667T>G(p.Leu556Arg) | NA | NA | Elevated | NA | NA | NA | NA | LT(8.5Y ) |  |
| 14 | Male | 3Y | Hepatomegaly, splenomegaly | c.1783C>T(p.Arg595*)/ c.937_992delGCACTGins55(p.Ala313Metfs*72) | Elevated | Elevated | Elevated | NA | NA | NA | Negative | Alive(5Y) | Giovannoni et al. 2011 |
| 15 | Female | 4Y | Jaundice, pruritus | c.1783C>T(p.Arg595*)/ c.1783C>T(p.Arg595*) | NA | NA | Elevated | NA | NA | NA | Negative | LT(7Y) |  |
| 16 | Male | 7M | Hepatomegaly, splenomegaly, pruritus, portal hypertension | c.67_68insAC(p.Leu23Hisfs16*)/ c.79A> G(p.Ser27Gly) | NA | NA | Elevated | Normal | NA | Elevated | Intermediate | Alive | Kubitz R 2011 |
| 17 | Female | 1.5M | Pruritus | c.286+1G>A/ c.3541C>G(p.Gln1181Glu) | NA | NA | Elevated | Normal | NA | NA | Positive | Alive |  |
| 18 | Male | 10M | Pruritus, jaundice, failure to thrive | c.3691C>T(p.His1231Tyr)/ c.3691C>T(p.His1231Tyr) | Elevated | Elevated | Elevated | Elevated | Elevated | Elevated | Intermediate | Alive(7Y) | Dzagania et al. 2012 |
| 19 | Female | 4.5Y | Jaundice, pruritus, hepatomegaly, splenomegaly, portal hypertension | c.1376A>G(p.Asp459Gly)/ c.3825_3826delA(p.Met1276Trpfs*33) | Elevated | Elevated | Elevated | Elevated | Elevated | Elevated | Intermediate | Alive(7Y) | Fang et al. 2012 |
| 20 | Female | 1M | Jaundice, pruritus, hepatomegaly, splenomegaly, failure to thrive, portal hypertension | c.344+2_+3insT/ c.2077_2078delC(p.Pro693Hisfs*6) | Elevated | Elevated | Elevated | Elevated | Elevated | Elevated | Intermediate | Alive(4Y) |  |
| 21 | Male | 3M | Jaundice, pruritus, hepatomegaly, splenomegaly, failure to thrive, portal hypertension | c.139C>T(p.Arg47*)/ c.1745G>A(p.Arg582Gln) | Elevated | Elevated | Elevated | Elevated | Elevated | Elevated | Intermediate | Alive(10Y) |  |
| 22 | Female | 11Y | Hepatomegaly, splenomegaly, ascites, gallstones | c.2563C>T (p.Gln855*)/ c.1283T>C(p.Val428Aal) | Elevated | Elevated | Elevated | NA | Normal | NA | Intermediate | Alive(17Y) | Ramraj et al. 2012 |
| 23 | Male | 6Y | Hepatomegaly, jaundice, splenomegaly | c.490T>G(p.Trp164Gly)/ c.3081+1 G>C | Elevated | Elevated | Elevated | NA | Elevated | NA | Intermediate | Alive(7.5Y) |  |
| 24 | Female | 15Y | Splenomegaly, jaundice | c.984T>C(p.Tyr328*)/ c.3218G>A(p.Cys1073Tyr) | Elevated | Elevated | Elevated | Elevated | Elevated | Elevated | Intermediate | Alive | Boga et al. 2015 |
| 25 | Female | 15M | Pruritus, hepatomegaly, failure to thrive | c.3559C>T(p.Arg1187*)/ c.3633+2T>A | Elevated | NA | Elevated | Elevated | NA | NA | Negative | LT(5Y) | Gordo-Gilart et al. 2015 |
| 26 | Female | 12M | Hepatomegaly, pruritus, splenomegaly | c.202G>A(p.Gly68Arg)/ c.202G>A(p.Gly68Arg) | Elevated | NA | Elevated | Normal | NA | NA | Negative | LT waiting list(6Y) |  |
| 27 | Female | 18M | Hepatomegaly, pruritus, jaundice | c.202G>A(p.Gly68Arg)/ c.202G>A(p.Gly68Arg) | Elevated | NA | Elevated | Elevated | NA | NA | Negative | LT(7.2Y) |  |
| 28 | Female | 23M | Pruritus, hepatomegaly, jaundice, gastrointestinal bleeding, splenomegaly | c.1375G>C(p.Asp459His)/ c.1436C>T(p.Pro479Leu) | Elevated | NA | Elevated | Elevated | NA | NA | Negative | LT waiting list(9.3Y) |  |
| 29 | Male | 8M | Hepatomegaly, pruritus | c.602C>T(p.Thr201Met)/ c.3352G>A(p.Glu1118Lys) | Elevated | NA | Elevated | Elevated | NA | NA | Positive | Alive(15Y) |  |
| 30 | Male | 15Y | Pruritus, jaundice, hepatomegaly, splenomegaly, portal hypertension | c.874A>T(p.Lys292*)/ c.1804G>T(p.Gly602Trp) | Elevated | Elevated | Elevated | Elevated | Elevated | Elevated | NA | Alive (17Y) | Sun et al. 2015 |
| 31 | NA | 1Y | Pruritus, hepatomegaly, portal hypertension | c.1270A>G(p.Thr424Ala)+c.2925-10_2925-9insC/  c.2925-10_2925-9insC | NA | NA | NA | NA | NA | NA | NA | LT(13Y) | Delaunay JL et al. 2016 |
| 32 | NA | 4Y | Pruritus, jaundice, hepatomegaly, portal hypertension | c.2860G>A(p.Gly954Ser)/ c.2860G>A(p.Gly954Ser) | NA | NA | NA | NA | NA | NA | NA | LT(21Y) |  |
| 33 | NA | 4Y | Pruritus, jaundice, hepatomegaly, portal hypertension | c.2860G>A(p.Gly954Ser)/ c.2860G>A(p.Gly954Ser) | NA | NA | NA | NA | NA | NA | NA | LT(21Y) |  |
| 34 | NA | 6M | Pruritus, hepatomegaly | c.523A>G(p.Thr175Ala)/ c.1069T>C(p.Phe357Leu)+c.2324C>T(p.Thr775Met) | NA | NA | NA | NA | NA | NA | NA | Alive(8.5Y) |  |
| 35 | NA | 15Y | Hepatomegaly | c.1529A>G(p.Asn510Ser)/ c.2177C>T(p. Pro726Leu) | NA | NA | NA | NA | NA | NA | NA | Alive(19Y) |  |
| 36 | NA | 1.5Y | Pruritus, hepatomegaly | c.2564A>T(p.Gln855Leu)/ c.2564A>T(p.Gln855Leu) | NA | NA | NA | NA | NA | NA | NA | Alive(25Y) |  |
| 37 | M | 3.5Y | Jaundice, hepatomegaly, splenomegaly, failure to thrive, abdominal distension, ascites | c.1006-2A>G/ c.3580C>T(p.Arg1194*) | Elevated | Elevated | Elevated | Elevated | Elevated | Elevated | NA | NA | Deng et al. 2018 |
| 38 | Female | 7Y | Asthenia, splenomegaly, jaundice | c.1436C>T(p.Pro479Leu)/ c.1436C>T(p.Pro479Leu) | Elevated | Elevated | Elevated | NA | NA | NA | Negative | LT(17Y) | Khabou et al. 2018 |
| 39 | Male | 7Y | Jaundice, abdominal distension, splenomegaly, gastrointestinal bleeding, portal hypertension | c.1436C>T(p.Pro479Leu)/ c.1436C>T(p.Pro479Leu) | Elevated | Elevated | Elevated | NA | NA | NA | Negative | Died(12Y) |  |
| 40 | Female | 9.5Y | Jaundice, splenomegaly, portal hypertension | c.1436C>T(p.Pro479Leu)/ c.1436C>T(p.Pro479Leu) | Elevated | Elevated | Elevated | NA | NA | NA | Negative | Died(10Y) |  |
| 41 | Female | 2Y | Pruritus, hepatomegaly, splenomegaly | c.1436C>T(p.Pro479Leu)/ c.1436C>T(p.Pro479Leu) | Elevated | Elevated | Elevated | NA | NA | NA | Intermediate | Alive(6Y) |  |
| 42 | Female | 1.5Y | Jaundice, pruritus, hepatomegaly | c.1436C>T(p.Pro479Leu)/ c.1436C>T(p.Pro479Leu) | Normal | Elevated | Elevated | NA | NA | NA | Intermediate | Alive(14Y) |  |
| 43 | Female | 1.5Y | Jaundice, pruritus, hepatomegaly | c.1436C>T(p.Pro479Leu)/ c.1436C>T(p.Pro479Leu) | Normal | Elevated | Elevated | NA | NA | NA | Intermediate | Alive(15Y) |  |
| 44 | Female | 5Y | Jaundice, hepatomegaly, splenomegaly, gastrointestinal bleeding | c.1436C>T(p.Pro479Leu)/ c.1436C>T(p.Pro479Leu) | Normal | Elevated | NA | NA | NA | NA | Negative | Died(13Y) |  |
| 45 | Female | 2.5Y | Hepatomegaly, splenomegaly | c.1565T>C(p.Phe522Leu)/ c.3859T>A(p.Ter1287Arg) | Elevated | Elevated | Elevated | Elevated | Elevated | NA | Negative | Alive | Küçükçongar Yavaş et al. 2020 |
| 46 | Male | 4Y | Hepatomegaly, splenomegaly, pruritus | c.2570C>T(p.Thr857Ile) +c.2212A>T(p.Ile738Phe)/  c.1694C>G(p.Thr565Arg) | Elevated | Elevated | Elevated | Normal | Normal | Elevated | NA | Alive | Li et al. 2020 |
| 47 | Male | 10M | Hepatomegaly, pruritus | c.2570C>T (p.Thr857Ile) +c.2212A>T(p.Ile738Phe)/  c.1694C>G (p.Thr565Arg) | Elevated | Elevated | Elevated | Normal | Normal | Elevated | NA | Alive |  |
| 48 | Female | 7Y | Jaundice, hepatomegaly, splenomegaly, ascites, cholecystitis, gallstone | c.1195G>( p.Val399Leu)/ c.1195G>( p.Val399Leu) | Elevated | Elevated | Elevated | Elevated | Elevated | NA | NA | Alive(13Y) | Saleem et al. 2020 |
| 49 | Male | 11M | Jaundice, pruritus, hepatomegaly, failure to thrive | c.2489insA(p.Thr830Asnfs*11)/ c.3139_3141delGCAinsCC(p.Ala1047Profs*8) | Elevated | NA | Elevated | Elevated | NA | Elevated | NA | NA | Zhang et al. 2020 |
| 50 | Female | 1.6Y | Jaundice, pruritus, hepatomegaly, splenomegaly, failure to thrive | c.955G>C(p.Gly319Arg)/ c.3220G>A(p.Gly1074Arg) | Elevated | NA | Elevated | Elevated | NA | Elevated | NA | NA |  |
| 51 | Male | 5Y | Pruritus, hepatomegaly, splenomegaly, failure to thrive | c.1436C>T(p.Pro479Leu)/ c.1436C>T(p.Pro479Leu) | Elevated | NA | Elevated | Normal | NA | Elevated | NA | Alive |  |
| 52 | Female | 4D | Jaundice, pruritus, hepatomegaly | c.3143delA(p.Asn1048Thrfs*8)/ c.3139G>C( p.Ala1047Pro) | Elevated | NA | Elevated | Elevated | NA | Elevated | NA | Died(10M) |  |
| 53 | Male | 6M | Pruritus, hepatomegaly | c.2692G>A(p.Glu898Lys)/ c.2692G>A(p.Glu898Lys) | Elevated | Elevated | Elevated | Normal | Normal | Elevated | Positive | Alive(10Y) | Al-Hussaini et al. 2020 |
| 54 | Female | 11M | Pruritus, hepatomegaly | c.2692G>A(p.Glu898Lys)/ c.2692G>A(p.Glu898Lys) | Elevated | Elevated | Elevated | Normal | NA | NA | Positive | Alive(7Y) |  |
| 55 | Female | 60M | Pruritus | c.2692G>A(p.Glu898Lys)/ c.2692G>A(p.Glu898Lys) | Elevated | Elevated | Elevated | Normal | NA | Elevated | Positive | Alive(11Y) |  |
| 56 | Male | 30M | Pruritus, hepatomegaly, splenomegaly | c.2692G>A(p.Glu898Lys)/ c.2692G>A(p.Glu898Lys) | Elevated | Elevated | Elevated | Normal | Normal | Elevated | Intermediate | Alive(8Y) |  |
| 57 | Male | 72M | Pruritus, hepatomegaly, recurrent epistaxis, coagulopathy | c.2692G>A(p.Glu898Lys)/ c.2692G>A(p.Glu898Lys) | Elevated | Elevated | Elevated | Normal | Normal | Elevated | Intermediate | Alive(9Y) |  |
| 58 | Female | 6M | Hepatomegaly, splenomegaly, failure to thrive, portal hypertension | c.2692G>A(p.Glu898Lys)/ c.2692G>A(p.Glu898Lys) | Elevated | Elevated | Elevated | Normal | Normal | Elevated | Intermediate | LT(9.5Y) |  |
| 59 | Male | 48M | Jaundice, pruritus, epistaxis, failure to thrive, portal hypertension | c.2692G>A(p.Glu898Lys)/ c.2692G>A(p.Glu898Lys) | Elevated | NA | Elevated | Normal | NA | Elevated | Intermediate | Alive(99M) |  |
| 60 | Male | 1M | Jaundice | c.2692G>A(p.Glu898Lys)/ c.2692G>A(p.Glu898Lys) | Normal | Elevated | Elevated | NA | NA | NA | Positive | Alive(10M) |  |
| 61 | Female | 10M | Pruritus, hepatomegaly, splenomegaly, portal hypertension | c.221T>G(p.Met74Arg)/ c.221T>G(p.Met74Arg) | Elevated | Elevated | Elevated | Normal | Normal | Elevated | Intermediate | LT(8Y) |  |
| 62 | Male | 24M | Pruritus, hepatomegaly | c.2906G>A(p.Arg969His)/ c.2906G>A(p.Arg969His) | Elevated | Elevated | Elevated | Normal | Normal | Elevated | Positive | Alive(10Y) |  |
| 63 | Male | 8M | Pruritus, hepatomegaly, failure to thrive | c.2906G>A(p.Arg969His)/ c.2906G>A(p.Arg969His) | Elevated | NA | Elevated | Normal | Normal | NA | Positive | Alive(11Y) |  |
| 64 | Female | 17M | Pruritus, hepatomegaly, splenomegaly, rickets | c.2906G>A(p.Arg969His)/ c.2906G>A(p.Arg969His) | Elevated | Elevated | Elevated | Normal | Normal | Elevated | Positive | Alive(14Y) |  |
| 65 | Male | 6M | Pruritus, hepatomegaly, splenomegaly | c.456G>A(p.Lys152Lys)/ c.1584G>C(p.Glu528Asp) | Elevated | Elevated | Elevated | Elevated | Elevated | Elevated | Positive | Alive(9Y) |  |
| 66 | Male | 1M | Pruritus, hepatomegaly | c.2908T>C(p.Phe970Leu)/ c.2908T>C(p.Phe970Leu) | Elevated | Elevated | Elevated | Normal | Normal | Elevated | Positive | Alive(15Y) |  |
| 67 | Female | 12M | Pruritus, hepatomegaly | c.526C>T(p.Arg176Trp)/ c.526C>T(p.Arg176Trp) | Elevated | Elevated | Elevated | Normal | Normal | Normal | Positive | Alive(15Y) |  |
| 68 | Female | 6M | Pruritus, hepatomegaly | c.526C>T(p.Arg176Trp)/ c.526C>T(p.Arg176Trp) | Elevated | NA | Elevated | Normal | Normal | NA | Positive | Alive(3Y) |  |
| 69 | Female | 6M | Pruritus, hepatomegaly, splenomegaly, portal hypertension | c.526C>T(p.Arg176Trp)/ c.526C>T(p.Arg176Trp) | Elevated | Elevated | Elevated | Normal | Normal | NA | Intermediate | LT(9Y) |  |
| 70 | Male | 6M | Pruritus, hepatomegaly | c.526C>T(p.Arg176Trp)/ c.526C>T(p.Arg176Trp) | Elevated | Elevated | Elevated | Normal | Normal | NA | Positive | Alive(3.5Y) |  |
| 71 | Male | 11M | Pruritus | c.296C>T(p.Ser99Phe)/ c.296C>T(p.Ser99Phe) | Elevated | Elevated | Elevated | Normal | Normal | Elevated | Intermediate | Alive(8.5Y) |  |
| 72 | Female | 18M | Pruritus | c.296C>T(p.Ser99Phe)/ c.296C>T(p.Ser99Phe) | Elevated | Elevated | Elevated | Normal | Normal | Normal | Positive | Alive(3Y) |  |
| 73 | Male | 11M | Pruritus, hepatomegaly, splenomegaly, portal hypertension | c.1378A>T(p.Ile460Phe)/ c.1378A>T(p.Ile460Phe) | Elevated | Elevated | Elevated | Elevated | Elevated | Elevated | Intermediate | LT(11Y) |  |
| 74 | Male | 1M | Pruritus, gastrointestinal bleeding, portal hypertension | c.2064+1G>C/ c.2064+1G>C | Normal | Elevated | Elevated | Elevated | Elevated | NA | Intermediate | Died(7Y) |  |
| 75 | Male | 2M | Failure to thrive, intracranial bleeding, hepatomegaly, portal hypertension | c.628_643del(p.Phe210Serfs*5)/ c.628_643de(p.Phe210Serfs*5) | Elevated | Elevated | Elevated | Elevated | Elevated | NA | Intermediate | LT(9Y) |  |
| 76 | Female | 18M | Pruritus, hepatomegaly, splenomegaly, gastrointestinal bleeding, portal hypertension | c.628_643del(p.Phe210Serfs*5)/  c.628_643de(p.Phe210Serfs*5) | Elevated | Elevated | Elevated | Elevated | Elevated | NA | Negative | LT(8Y) |  |
| 77 | Male | 18M | Pruritus, hepatomegaly, splenomegaly, ascites, portal hypertension | c.628_643del(p.Phe210Serfs*5)/  c.628_643de(p.Phe210Serfs*5) | Elevated | Elevated | Elevated | Elevated | Elevated | NA | Negative | LT(6Y) |  |
| 78 | Male | 14Y | Jaundice, pruritus, splenomegaly | c.2525T>C(p.Leu842Pro)/ c.3152T>C(p.Val1051Ala) | Elevated | Elevated | Elevated | Elevated | Elevated | Elevated | NA | Alive | Bai et al. 2021 |
| 79 | Male | 6Y | Hepatomegaly, splenomegaly, jaundice, , gallstone | c.2149T>A(p.Cys717Ser)/ c.1745G>A(p.Arg582Gln) | Elevated | Elevated | Elevated | Normal | Normal | Elevated | NA | LT(19Y) | Lipiński et al. 2021 |
| 80 | Female | 2.5Y | Pruritus, hepatomegaly, splenomegaly, jaundice | c.3524T>A(p.Leu1182His)/ c.3524T>A(p.Leu1182His) | Elevated | Elevated | Elevated | Normal | Normal | Elevated | NA | LT(12Y) |  |
| 81 | Male | 14Y | Hepatomegaly, splenomegaly | c.959C>T(p.Ser320Phe)/ c.1119+1G>A | Elevated | Elevated | Elevated | Normal | Normal | Elevated | Intermediate | Alive(18Y) |  |
| 82 | Male | 2Y | Pruritus, hepatomegaly, splenomegaly | c.902T>A(p.Met301Lys)/ c.3279+1G>A | Elevated | Elevated | Elevated | Normal | Normal | Elevated | Intermediate | Alive(3.5Y) |  |

Y, year; M, month; D, day; NA, not available; LT, liver transplantation; UDCA: ursodeoxycholic acid; Positive response of UDCA: normalization of liver tests; Negative response of UDCA: no improvement; Intermediate response of UDCA: reduction of liver enzymes without reaching normal values or with ongoing symptoms.
